# *cat*RAPID *omics*: a web server for large-scale prediction of protein–RNA interactions

**DOI:** 10.1093/bioinformatics/btt495

**Published:** 2013-08-23

**Authors:** Federico Agostini, Andreas Zanzoni, Petr Klus, Domenica Marchese, Davide Cirillo, Gian Gaetano Tartaglia

**Affiliations:** ^1^Gene Function and Evolution, Bioinformatics and Genomics, Centre for Genomic Regulation (CRG) and ^2^Universitat Pompeu Fabra (UPF), 08003 Barcelona, Spain

## Abstract

**Summary:** Here we introduce *cat*RAPID *omics, a* server for large-scale calculations of protein–RNA interactions. Our web server allows (i) predictions at proteomic and transcriptomic level; (ii) use of protein and RNA sequences without size restriction; (iii) analysis of nucleic acid binding regions in proteins; and (iv) detection of RNA motifs involved in protein recognition.

**Results:** We developed a web server to allow fast calculation of ribonucleoprotein associations in *Caenorhabditis elegans, Danio rerio, Drosophila melanogaster, Homo sapiens, Mus musculus, Rattus norvegicus, Saccharomyces cerevisiae* and *Xenopus tropicalis* (custom libraries can be also generated)*.* The *cat*RAPID *omics* was benchmarked on the recently published RNA interactomes of Serine/arginine-rich splicing factor 1 (SRSF1), Histone-lysine N-methyltransferase EZH2 (EZH2), TAR DNA-binding protein 43 (TDP43) and RNA-binding protein FUS (FUS) as well as on the protein interactomes of U1/U2 small nucleolar RNAs, X inactive specific transcript (Xist) repeat A region (RepA) and Crumbs homolog 3 (CRB3) 3′-untranslated region RNAs. Our predictions are highly significant (*P* < 0.05) and will help the experimentalist to identify candidates for further validation.

**Availability:** *cat*RAPID *omics* can be freely accessed on the Web at http://s.tartaglialab.com/catrapid/omics. Documentation, tutorial and FAQs are available at http://s.tartaglialab.com/page/catrapid_group.

**Contact:** gian.tartaglia@crg.eu

## 1 INTRODUCTION

Increasing evidence indicates that ribonucleoprotein interactions are fundamental for cellular regulation (Khalil and Rinn, 2011). Moreover, several studies highlighted the involvement of RNA molecules in the onset and progression of human diseases including neurological disorders (Johnson *et al.*, 2012). To our knowledge, there are two sequence-based methods for prediction of protein–RNA interactions: *cat*RAPID (Bellucci *et al.*, 2011) and RPISeq (Muppirala *et al.*, 2011). The *cat*RAPID algorithm exploits predictions of secondary structure, hydrogen bonding and van der Waals’ contributions to estimate the binding propensity of protein and RNA molecules. RPISeq is based on support vector machine (SVM) and random forest (RF) models predicting protein–RNA interactions from primary structure alone (Muppirala *et al.*, 2011). Both methods show remarkable performances, but *cat*RAPID discriminates positive and negative cases with higher accuracy (Cirillo *et al.*, 2013b) and has been tested on long non-coding RNAs (Agostini *et al.*, 2013).

Here we introduce *cat*RAPID *omics* to perform high-throughput predictions of protein–RNA interactions using the information on protein and RNA domains involved in macromolecular recognition.

## 2 WORKFLOW AND IMPLEMENTATION

The *cat*RAPID *omics* server provides two main services to explore the interaction potential of (i) a protein of interest with respect to a target transcriptome or (ii) a given RNA with respect to the nucleic acid binding proteome. Several options are available to refine the type of analysis in eight model organisms or custom libraries (see online documentation):

- In the case of a protein query, *cat*RAPID *omics* takes as input the protein sequence (FASTA format): full-length or, alternatively, nucleic acid binding regions.
- For a transcript query (FASTA format), the server uses the full-length sequence if below 1200 nt, or, alternatively, uses fragments with predicted stable secondary structure (Agostini *et al.*, 2013). Full-length proteins and nucleic acid binding regions can be searched.
- The server automatically detects disordered proteins lacking canonical RNA binding domains. Indeed, it has been observed that disordered regions are enriched in RNA binding proteins (Castello *et al.*, 2012).
- As RNA motifs are important for protein recognition (Kazan *et al.*, 2010), a search for these elements is carried out. The motifs were taken from RNA-Binding Protein DataBase (RBPDB) (Cook *et al.*, 2011), SpliceAid-F (Giulietti *et al.*, 2013) and a recent motif compendium (Ray *et al.*, 2013).
- Using the interaction propensities distribution, *cat*RAPID *omics* predicts the RNA binding ability of the input protein (86% accuracy) and ranks RNA interactions (downloadable by the user).

**Fig. 1.**
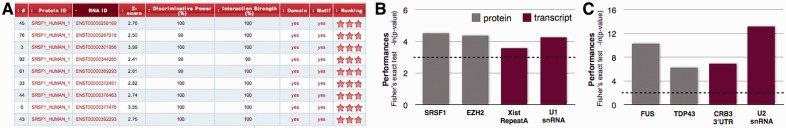
*cat*RAPID *omics* features and performances. (**A**) Example of the output table showing *Z*-score (interaction propensity normalized with respect to experimental cases), discriminative power (with respect to training sets), interaction strength (enrichment with respect to random interactions) and presence of RNA binding domains as well as RNA motifs. Interaction scores are ranked according to a ‘star rating system’ ranging from 0 to 3 (http://service.tartaglialab.com/static_files/shared/faqs.html). A click on the text redirects to reference pages. Performances on (**B**) full-length proteins and (**C**) RNA binding protein domains. Gray is used to highlight transcriptomic studies (i.e. RNA sequencing) and red indicates proteomic analyses (i.e. mass spectrometry). The significance of our predictions was assessed using Fisher’s exact test (the dashed line corresponds to *P* = 0.05)

In the output page (Fig. 1A), we report all the variables used to estimate protein–RNA associations: interaction propensity (Bellucci *et al.*, 2011), discriminative power (Bellucci *et al.*, 2011), interaction strength (Agostini *et al.*, 2013) and presence of protein RNA binding domains as well as RNA motifs. A ‘star rating system’ ranks the binding propensities (http://service.tartaglialab.com/static_files/shared/faqs.html). As for the reference sets, ENSEMBL (version 68) is used for retrieval and classification of coding and non-coding RNAs, whereas protein sequences are gathered from the UniProtKB database (release 2012_11). Finally, *cat*RAPID *omics* uses hmmscan, a Hidden Markov Model-based algorithm from the HMMER3 package (Finn *et al.*, 2011), to identify known PfamA domains (Finn *et al.*, 2009) and recognize protein regions involved in binding nucleic acid molecules. Algorithm hit significance is determined according to the PfamA ‘gathering thresholds’.

## 3 PERFORMANCES

The *cat*RAPID algorithm has been previously validated on a number of protein–RNA associations (Agostini *et al.*, 2013; Bellucci *et al.*, 2011; Cirillo, *et al.*, 2013a; Johnson *et al.*, 2012). To evaluate large-scale performances of *cat*RAPID *omics*, we used data from recent large-scale experiments. To compare predicted and experimental interactions, we used Fisher’s exact test. As shown in Figure 1B, performances on the human splicing factor serine/arginine-rich splicing factor 1 (SRSF1) (Sanford *et al.*, 2009) and murine nucleic acid binding protein Histone-lysine N-methyltransferase EZH2 (EZH2) (Zhao *et al.*, 2010) are highly significant (*P*-values: 0.01 and 0.01, respectively). Good performances are found for low-throughput experiments on murine non-coding X inactive specific transcript (Xist) repeat A region (RepA) (Maenner *et al.*, 2010; Royce-Tolland *et al.*, 2010) and yeast small nuclear RNA U1 (Cvitkovic and Jurica, 2012) (*P*-values: 0.03 and 0.015) (Fig. 1B). To illustrate the ability of *cat*RAPID *omics* to predict interactions with nucleic acid binding domains (Fig. 1C), we used murine FUS (Han *et al.*, 2012) and rat TAR DNA-binding protein 43 (TDP43) (Sephton *et al.*, 2011) (*P*-values: 3e-05 and 0.002) as well as human Crumbs homolog 3 (CRB3) 3′-untranslated region (Iioka *et al.*, 2011) and yeast small nuclear U2 (Cvitkovic and Jurica, 2012) (*P*-values: 0.001 and 2e-0.6). To evaluate *cat*RAPID’s performances on high-throughput data, we collected positive interactions (TDP43: 568, FUS: 99, SRSF1: 358, EZH2: 1141) as well as negative controls (same numbers as positives and generated in four random extractions). Comparing the interaction scores of positives and negatives, we found enrichment (calculated as discriminative power) in 72% (TDP43), 88% (FUS), 74% (SRSF1) and 56% (EZH2) of cases. On the same datasets, SVM RPIseq showed enrichment in 58% (TDP43; RF has enrichment in 53%), 83% (FUS; RF has enrichment in 68%), 47% (SRSF1; RF has enrichment in 59%) and 41% (EZH2; RF has enrichment in 48%) of cases. 

## 4 CONCLUSIONS

Despite recent technical developments, detection of protein–RNA associations remains a challenging task. For this reason, we developed an algorithm that can be used to complement experimental efforts (Zanzoni *et al.*, 2013). The *cat*RAPID *omics* server offers unique features such as organism-specific proteomic and transcriptomic libraries, possibility to generate custom datasets, analysis of long sequences and calculation of interaction specificities. Moreover, we implemented an algorithm for the detection of RNA motifs as well as protein RNA binding domains, which will help to retrieve recognition motifs embedded in sequences. Our server enables fast calculations of ribonucleoprotein associations and predicts RNA binding activity of proteins with high accuracy, thus resulting in a powerful tool for designing new experiments.
